# Update on umbilical cord blood transplantation

**DOI:** 10.12688/f1000research.11952.1

**Published:** 2017-08-24

**Authors:** Karen Ballen

**Affiliations:** 1 Stem Cell Transplant Program, University of Virginia Health System, Charlottesville, VA, USA

**Keywords:** Allogeneic hematopoietic cell transplantation, Umbilical cord blood transplantation, Regenerative Medicine

## Abstract

Allogeneic hematopoietic cell transplant is a curative procedure for many patients with leukemia, lymphoma, myelodysplasia, myeloproliferative neoplasms, and genetic disorders. Umbilical cord blood transplantation is a graft source for patients who do not have a matched donor in their family or in the unrelated registry. It is particularly difficult for Black, Hispanic, and White patients of non-Western European background to find fully matched adult volunteer donors. An estimated 700,000 umbilical cord blood units have been donated for public use, and over 40,000 umbilical cord blood transplantations have been performed. Over 25,000 patients have been cured with this approach.

##  Introduction

Traditionally, allogeneic hematopoietic cell transplant (HCT) has been limited to fully matched related or unrelated donors. Each brother or sister has a 25% chance of matching the patient; given the size of most families in the US and Western Europe, only 30% of patients will have a fully matched donor in their family ^1^. Because the cells from the newborn baby are immunologically more naïve, there is less risk of the immune-mediated complication of graft-versus-host disease after umbilical cord blood transplantation (UCBT) and therefore the patient and the UCB unit do not need to be as closely matched. Thus, patients of diverse racial/ethnic background may be more likely to find a suitable UCB donor when they cannot find a family or registry donor ^2^. In this overview, we discuss cord blood banking, pediatric and adult UCBT, and future directions in the field. We compare UCBT with other transplant approaches, including haploidentical (half-matched) transplant.

## Cord blood banking

The UCB unit is collected from term healthy babies. It can be collected after vaginal or surgical deliveries. UCB collection does not interfere in any way with the normal delivery process. Recently, there has been more interest in delayed cord blood clamping. The American College of Obstetrics and Gynecology and other professional organizations have issued position statements on the recommended cord blood clamping time, which collectors of UCB are urged to follow ^3^.

In 2011, the US Food and Drug Administration began licensure of UCB units, and currently less than 10% of the worldwide UCB inventory is licensed. Unlicensed UCB units can be obtained under an Investigational New Drug Application, and outcome results are excellent with these UCB units as well ^4^. Cord blood banking practices have changed over the years to more automated methods, with no impact on survival ^5^.

## Pediatric umbilical cord blood transplants

The first successful related and unrelated donor UCBTs were performed in children ^6, 7^. Although there have been no randomized prospective studies comparing outcomes among graft sources, several retrospective studies have shown comparable survival ^8^. Outcomes for pediatric patients with acute leukemia receiving unrelated UCBT were compared with outcomes after a matched unrelated donor transplant (HCT) ^9^. The best results were in children who received fully matched UCBT. Results were comparable between traditional unrelated bone marrow transplantation and UCBT. Single versus double UCBT has been studied in a prospective randomized study and there was no advantage to the most costly double UCBT ^10^. Children with metabolic disorders such as Hurler syndrome, Krabbe disease, and Sanfilippo syndrome have excellent outcomes if transplanted early in their disease course (75% at 5 years) ^11^.

## Adult cord blood transplantation

After the initial encouraging results in children, UCBT was extended to adults with hematologic malignancies who did not have a matched family or volunteer donor. Initial results indicated a high transplant-related mortality, which improved with the use of better UCB unit selection, modern supportive care, especially regarding infection prevention and treatment, and the use of UCB units with higher cell doses ^12^. Double UCBT (using two partially matched UCB units) and the use of reduced-intensity conditioning were pioneered by the group at the University of Minnesota and incorporated into several transplant centers, with disease-free survival of 35–45% ^13– 15^. The use of single versus double UCBT has not been tested in a phase III study in adults and remains controversial.

FMS-like tyrosine kinase (FLT3) acute myeloid leukemia has a high risk of relapse and is an indication for HCT. Leukemia-free survival was similar among patients who received UCBT, sibling HCT, or matched unrelated donor, although graft-versus-host disease was lowest in UCBT ^16^.

Selection of the optimal UCB unit is more complex than in standard related donor or unrelated donor transplantation. In addition to HLA matching, there are decisions regarding cell dose, HLA antibodies, and cell viability. Patients who harbor donor-specific HLA antibodies against the chosen UCB unit have been shown to have poorer engraftment and survival, and these units should be avoided ^17^.

## Comparison with haploidentical transplantation

A haploidentical HCT is an HCT from a half-matched donor. Based on genetics, children, parents, and 50% of siblings could serve as a haploidentical donor. Parallel phase 2 studies showed similar 1-year overall and progression-free survivals ^18^. A large national US randomized, phase III study comparing these two graft sources is under way. This is a high-priority study for the transplant community.

## New trends in cord blood transplantation

One of the limiting factors of UCBT is the delayed engraftment and immune recovery that may lead to infection, particularly uncommon viral infections ^19^. Leukemia patients with a poor performance status have decreased survival with UCBT compared with other graft sources, likely due to high rates of infection and transplant-related complications. Several strategies have been undertaken to improve this delayed immune recovery: homing to the bone marrow, *ex vivo* expansion, and infection prophylaxis ( Table 1) ^20^. All studies are small and none has documented improved survival in a randomized study. In general, the *ex vivo* expansion studies require more specialized techniques available in only a few centers. However, the cost of procurement of each UCB unit is $30,000 to $45,000, which may make *ex vivo* expansion of 1 UCB unit more cost-effective than using 2 UCB units. Homing strategies include intrabone marrow UCBT, in which the UCB unit is injected directly into the iliac crest. A Japanese study showed faster platelet engraftment and improved donor chimerism (the measure of donor versus recipient DNA) with this approach ^21^. Selectins are needed to initiate stem cell homing and are modified via a fucosylation process. A small study using fucosylation of UCB cells has shown engraftment in 14 days ^22^. The combination of haploidentical with either a single or double UCBT has been shown to improve engraftment ^23– 25^.

**Table 1.** Novel strategies to improve engraftment.

Strategy	Mechanism	Investigators	Number	Days to absolute neutrophil count > 500
Intrabone marrow injection	Homing	Kurita *et al*. ^21^	15	7
Nicotinamide	Expansion	Horwitz *et al*. ^26^	11	13
Fucosylation	Homing	Popat *et al*. ^22^	7	14
Notch	Expansion	Delaney *et al*. ^27^	10	16

*Ex vivo* expansion studies include the use of the Notch Ligand, Delta 1, mesenchymal progenitor cell expansion, and a nicotinamide-based expansion with cytokines ^26– 28^. This last approach resulted in engraftment in 13 days and now is part of a randomized controlled trial.

One novel approach to decrease viral infection is to use tri virus (adenovirus, Epstein-Barr virus, and cytomegalovirus)–expanded T cells. These T cells have been used successfully to treat refractory viral infections after UCBT ^29^.

## Regenerative medicine

An exciting new opportunity is using either autologous or unrelated UCBT for diseases outside the traditional scope of oncology. UCB stem cells have greater proliferative potential than adult bone marrow stem cells ^30^. UCB has been used to treat neurologic diseases such as autism, cerebral palsy, hypoxic ischemic encephalopathy, and traumatic brain injury ^31^. In cerebral palsy, intravenous autologous UCB infusions have been administered safely ^32^. Allogeneic infusions have also been used; 47 patients with severe cerebral palsy received safe treatment with unmatched allogeneic UCB cells, given both intravenously and intrathecally ^33^. Gross motor function scores improved, and there was no graft-versus-host disease ^34^.

In cardiovascular disease, several trials are under way for cardiomyopathy and coronary artery disease. In cardiovascular disease, UCB mesenchymal stem cells secrete cytokines that stimulate angiogenesis ^35^. In animal models (rat) of myocardial infarction, UCB-derived mesenchymal stem cells have demonstrated decreased size of myocardial infarct and improved cardiac pumping function ^36, 37^.

About 15 million babies are born preterm worldwide; owing to hypoxia-ischemia, these babies are at high risk of neurodevelopmental problems ^38^. Clinical trials using UCB are ongoing at Duke University Medical Center and the National University of Singapore (ClinicalTrials.gov Identifier: NCT00593242) ^31, 39^. Human UCB-derived cells are also being investigated for the treatment of inflammatory bowel disease, corneal disease, renal disease, and collagen-induced arthritis ^40– 42^.

## Conclusions

From the first UCBT in 1988, the field of UCBT has evolved considerably ^43^. UCBT is now a standard treatment for both children and adults who do not have matched sibling or unrelated donors. A large clinical trial is under way to compare UCBT with haploidentical HCT. Transplant outcomes continue to improve with refinement in UCB unit selection and infection prevention. *Ex vivo* expansion and homing strategies are in clinical trials to reduce the risk of infection. Finally, new applications of UCB in cerebral palsy, autism, and cardiovascular disease are likely to make major health impacts in the next 5–10 years.
